# HiCancer: accurate and complete cancer genome phasing with Hi-C reads

**DOI:** 10.1038/s41598-021-86104-6

**Published:** 2021-03-23

**Authors:** Weihua Pan, Desheng Gong, Da Sun, Haohui Luo

**Affiliations:** Shenzhen Branch, Guangdong Laboratory for Lingnan Modern Agriculture, Genome Analysis Laboratory of the Ministry of Agriculture, Agricultural Genomics Institute at Shenzhen, Chinese Academy of Agricultural Sciences, Shenzhen, 518120 China

**Keywords:** Cancer genomics, Software, Computational biology and bioinformatics, Computational models

## Abstract

Due to the high complexity of cancer genome, it is too difficult to generate complete cancer genome map which contains the sequence of every DNA molecule until now. Nevertheless, phasing each chromosome in cancer genome into two haplotypes according to germline mutations provides a suboptimal solution to understand cancer genome. However, phasing cancer genome is also a challenging problem, due to the limit in experimental and computational technologies. Hi-C data is widely used in phasing in recent years due to its long-range linkage information and provides an opportunity for solving the problem of phasing cancer genome. The existing Hi-C based phasing methods can not be applied to cancer genome directly, because the somatic mutations in cancer genome such as somatic SNPs, copy number variations and structural variations greatly reduce the correctness and completeness. Here, we propose a new Hi-C based pipeline for phasing cancer genome called HiCancer. HiCancer solves different kinds of somatic mutations and variations, and take advantage of allelic copy number imbalance and linkage disequilibrium to improve the correctness and completeness of phasing. According to our experiments in K562 and KBM-7 cell lines, HiCancer is able to generate very high-quality chromosome-level haplotypes for cancer genome with only Hi-C data.

## Introduction

Human genomes are diploid with two homologous sets of chromosomes. Each pair of homologous chromosomes share high similarity but are different in genetic variants such as single nucleotide polymorphism’s (SNPs) and insertions/deletions. The sequences of the variants on single chromosomes are called haplotypes, which provide us with more information of genetic makeup in an individual genome. The reconstruction of haplotypes, called phasing, plays important roles in different areas of biology such as genome-wide association^1–3^ and population genetics studies^4,5^ and is critical for advancing precision medicine^6,7^. Due to the extensive somatic variations such as somatic SNPs, structural variations (SVs) and copy number variations (CNVs), cancer genome is much more complicated than normal human genome. Although the complete map of cancer genome should contain the sequence of every single DNA molecule, with the existing technologies, it is believed too difficult to distinguish the copies of chromosomes on the same allele (from the same parent) generated by CNVs according to somatic SNPs. Nevertheless, phasing each cancer chromosome into two haplotypes according to germline mutations as done for normal genome provides a suboptimal solution to understand cancer genome.

However, phasing cancer genome is also a challenging problem, due to the limit in experimental and computational technologies. As far as we know, among the widely used cancer cell lines, only K562 and Hela have comparatively high quality chromosome-level phased genome available which are constructed by integrating multiple types of data^8,9^. The existing technologies of phasing can be divided into three main categories. The trio-based methods such as TetraOrigin^10^ and Merlin^11^ generate the haplotypes which are most consistent with pedigree structure and Mendelian segregation. These methods work well when the parent-child relationship is available. The population-based methods such as Beagle^12^ and SHAPTIT^13^ estimate haplotypes of an individual genome through linkage disequilibrium measures learned from a population of unrelated known haplotypes. These methods can accurately infer haplotypes up to ~300 kb, but are not able to generate chromosome-level haplotypes^14^. The sequencing-baseed methods such as HapTree^15^ and HapCompass^16^ take advantage of sequenced long reads or paired-end short reads to link variants into haplotypes directly. Compared with the trio-based and population-based methods which use extra information to estimate haplotypes, the sequencing-based methods take advantage of the information (reads) directly obtained from the genome to be phased, and thus are able to generate more reliable haplotypes and have a broader range of applications. Nevertheless, due to the lack of long-range linkage information for linking distant SNPs, it is still difficult for the traditional sequencing-based methods to generate chromosome-level haplotypes for normal human genome with only one type of sequence information, not to mention cancer genome.

The Hi-C based methods are a new type of sequencing-based methods which provide an opportunity to solve the problem of cancer genome phasing. Hi-C technology was originally developed for mapping the spatial structure of genome. In recent years, Hi-C paired-end reads have been widely used in phasing, due to the lower cost than long reads and the much longer-range linkage information. For instance^14^, applied Hi-C reads to phase human genome and mouse genome for the first time. And^17^ improved a phasing tool HAPCUT^18^ into version 2 by enhancing its performance on Hi-C reads . However, the existing Hi-C based phasing methods like HAPCUT2 can not be applied to cancer genome directly for at least two reasons. First, cancer specific genome features such as somatic mutations and loss of heterozygosity (LOH) may reduce the correctness of phasing if not specially dealt with. Specifically, LOH regions are believed to lose one of the two alleles and will be phased into two haplotypes by mistake if treated same as other regions. Also, somatic SNPs will disturb phasing if treated as germline SNPs. Second, due to the influence of CNVs and SVs, the haplotypes are generated with low completeness. For example, according to our experiments, when phasing K562 (human immortalized chronic myeloge-nous leukemia (CML) cell line) genome, HAPCUT2 lost about 20% of the SNPs and nearly all LOH regions in the genome, and the haplotypes contain switching errors in the middle of some chromosomes.

In this paper, we propose a new Hi-C based pipeline for phasing cancer genome called HiCancer, using HAPCUT2 as a module inside. HiCancer uses Hi-C paired-end reads and called SNPs as input and outputs chromosome-level haplotypes of cancer genome. HiCancer filters somatic SNPs and phase the LOH regions in a correct way. At the same time, HiCancer takes advantage of allelic copy number imbalance in aneuploid regions and linkage disequilibrium information to improve the completeness and accuracy by assembling fragmented haplotypes, adding the lost SNPs back into haplotypes (imputation) and correcting the switching errors. According to our experiments on K562 cell line, HiCancer significantly improves the phasing performance of HAPCUT2 on cancer genome in both completeness and correctness. We believe that HiCancer is able to generate high quality cancer haplotypes using only Hi-C data .

## Methods

**Figure 1:**
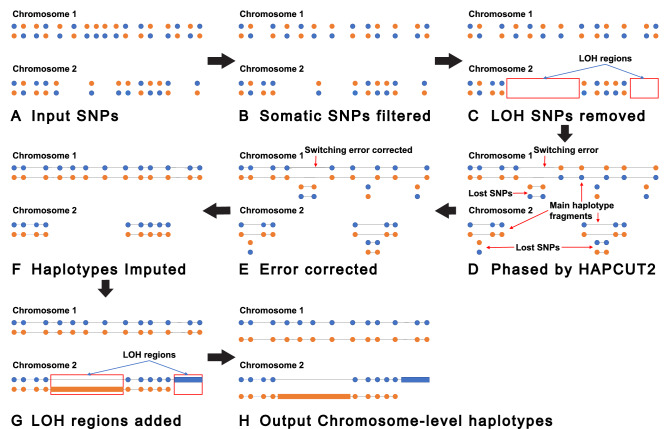
Pipeline of HiCancer. (**A**) The input unphased SNPs. Each blue-orange pair represents the two alleles of one SNP. Blue and orange represent two haplotypes of each chromosome. (**B**) Somatic SNPs are detected and removed from the input SNPs. (**C**) LOH regions are detected and SNPs in LOH regions are removed. (**D**) Pre-processed SNPs in non-LOH regions are phased by HapCut2. After phasing, in addition to main haplotype fragments, a large number of small fragments with one or a few SNPs exist. And main haplotype fragments contain some switching errors. (**E**) Switching errors are corrected. (**F**) The small haplotype fragments are merged into main haplotype fragments. (**G**) The sequences in LOH regions and the haplotyes in non-LOH regions are connected to chromosome-level haplotypes. (**H**) The final output chromosome-level haplotypes.

HiCancer is composed of four steps: pre-phasing processing, phasing, post-phasing processing and completing. In the pre-phasing processing, somatic SNPs are filtered, and the genome is divided into LOH regions and non-LOH regions. In the phasing step, non-LOH regions are phased into haplotypes. In the post-phasing processing step, a series of strategies are used to improve the completeness and correctness of haplotypes. In the completing step, sequences in LOH regions and phased haplotypes in non-LOH regions are assembled into the chromosome-level haplotypes. The pipeline of the proposed method is illustrated in Fig. 1.

### Step1–2: pre-phasing processing and phasing

In the pre-phasing processing step, we filter somatic SNPs and detect LOH regions in the genome. The SNPs not appearing in the SNP list provided by 1000 Genomes Project Phase 3^19^ are seen as somatic SNPs and removed. LOH regions are detected by comparing the density of heterozygous SNPs on the cancer genome with that on a normal genome in the same region. Specifically, the human genome is partitioned into segments of fixed length (typically 1 Mbp) and a statistical test is used on each segment to decide whether it is a LOH region on cancer genome. For each segment, we obtain the proportions of heterozygous and homozygous SNPs $p_{hetero}^{normal}$ and $p_{homo}^{normal}=1-p_{hetero}^{normal}$ from a normal cell line GM12878. Given the total number of SNPs *N* on cancer genome in the same region fixed, the number of heterozygous SNPs $N_{hetero}^{cancer}$ is assumed to be binomial distributed with parameters *N* and $p_{hetero}^{normal}$. With one-tailed test carried out, this region will be recognized as LOH region if p-value is smaller than some threshold (typically 0.05), which means the proportion of heterozygous SNPs in this region on cancer genome significantly lower than that on normal genome. The SNPs in the recognized LOH regions are seen as false positive and removed before phasing.

Next, the Hi-C reads are aligned to the human reference genome with BWA mem^20^ and the alignments uniquely aligned with MAPQ higher than some threshold (e.g, 30) are kept. Specifically, the paired-end reads are split into two groups, one for each pair, and the two groups are aligned separately and then combined by a Perl script “two_read_bam_combiner.pl” downloaded from https://github.com/dixonlab/bwa_mem_hic_aligner. Then, the SNPs in each continuous non-LOH region are phased by HAPCUT2 with default parameters using Hi-C paired-end reads. According to our experiments, the phased haplotypes of each continuous non-LOH regions usually contain one or a few main haplotype fragments and a large number of tiny fragments with one or more SNPs (see Fig. 1D). And some haplotypes contain fatal switching errors (see Fig. 1D,E).

### Step3: post-phasing processing

Due to the CNVs, many regions on cancer genome are aneuploid regions with different copy numbers on two alleles, leading to different read coverages between alleles after mapping Hi-C reads. According to our observations, when assembling two adjacent phased haplotype fragments, the haplotypes with similar read coverages are more likely on the same allele. In the post-phasing processing step, this regulation is used to improve the completeness and correctness of the phased haplotypes. First, the allelic read coverage imbalance are leveraged to correct the switching errors. Second, the allelic read coverage imbalance and linkage disequilibrium information are used to assemble the fragmented haplotypes and fill the gaps in haplotypes by adding the lost SNPs back into haplotypes. We describe these strategies in detail as follows.

#### Switching error correction

We search for the potential switching points on the haplotypes of each non-LOH segment. At each position between adjacent SNPs, four counts $c_1^{before}$, $c_1^{after}$, $c_2^{before}$ and $c_2^{after}$ are obtained. $c_1^{before}$ represents the number of SNPs before this position whose read coverage on haplotype $h_1$ is higher than haplotype $h_2$ and the other three counts have similar meanings correspondingly. With these counts, two ratios $r^{before}=c_1^{before}/c_2^{before}$ and $r^{after}=c_1^{after}/c_2^{after}$ can be obtained. We define a position as a potential switching point if the two ratios are significantly different. In detail, if one of these two ratios is larger than some threshold (e.g, 2) and at the same time the other one is smaller than some threshold (e.g, 0.5), this position is seen as a potential switching point. Since usually many qualified potential switching points exist, we pick the one with the lowest ratio of ratios

$$\begin{aligned} r^{r}=\frac{min\{r^{before}, r^{after}\}}{max\{r^{before}, r^{after}\}} \end{aligned}$$

and switch the haplotypes at it. This process is repeated until no potential switching point can be found.

Dynamic programming vectors are used to obtain counts for all positions efficiently. Let $c_1^{before}[i]$ be the number of SNPs whose read coverage on haplotype $h_1$ higher than haplotype $h_2$ before *i*th SNP. First we initialize $c_1^{before}[0]$ to be 0. The rest part of the dynamic programming vector can be filled using the following recurrence relation:

$$\begin{aligned} c_1^{before}[i] =c_1^{before}[i-1]+ \mathbb {1}\{cov_1[i] > cov_2[i]\} \end{aligned}$$

where $cov_1[i]$ and $cov_2[i]$ represent read coverages of *i*th SNPs on haplotype $h_1$ and haplotype $h_2$ respectively. The other counts can be calculated in the similar way with the following recurrence relations:

$$\begin{aligned} c_1^{after}[i]= & {} c_1^{after}[i+1]+ \mathbb {1}\{cov_1[i] > cov_2[i]\} \\ c_2^{before}[i]= & {} c_2^{before}[i-1]+ \mathbb {1}\{cov_1[i]< cov_2[i]\} \\ c_2^{after}[i]= & {} c_2^{after}[i+1]+ \mathbb {1}\{cov_1[i] < cov_2[i]\}. \end{aligned}$$

#### Assembly of haplotype fragments and gap filling

We developed a graph model called coverage matching graph (CMG) to assemble main haplotype fragments and fill the gaps with tiny haplotype fragments at the same time. A CMG *G* is an undirected weighted graph in which each vertex represents one of the two haplotypes of a single SNP. Given two SNPs $$s_i$$ and $$s_j$$ belonging to different fragments with haplotypes $$s_i^a$$, $$s_i^b$$ and $$s_j^a$$, $$s_j^b$$ respectively, we create four undirected edges $$(s_i^a, s_j^a)$$, $$(s_i^a, s_j^b)$$, $$(s_i^b, s_j^a)$$, $$(s_i^b, s_j^b)$$ in *G* if (1) the genomic distance between $$s_i$$ and $$s_j$$ does not exceed some threshold (e.g, 1 Mbp); (2) $$s_i$$ is one of the *n* (e.g, 5) closest SNPs to $$s_j$$ and $$s_j$$ is same to $$s_i$$; (3) at least one of $$s_i$$ and $$s_j$$ belong to the the block exceeding a minimum number of SNPs (e.g, 100) and (4) the read coverage on each of $$(s_i^a$$, $$s_i^b)$$, $$(s_j^a$$ and $$s_j^b)$$ is higher than some threshold (e.g, 10).

Each of these four edges is assigned a weight $w(s_i^x, s_j^y)$ which represents the likelihood of the two vertices connected belonging to the same haplotype. In detail, the calculation of edge weights is based on the probabilistic model below. Let’s take $w(s_i^a, s_j^a)$ and $w(s_i^b, s_j^b)$ which represent the likelihood of haplotypes “*aa*|*bb*” for instance.

Given the read coverages $c(s_i^a)$ and $c(s_i^b)$ of $s_i^a$, $s_i^b$, the read coverage proportions can be calculated as $p_i^a = c(s_i^a) / (c(s_i^a) + c(s_i^b))$ and $p_i^b = c(s_i^b) / (c(s_i^a) + c(s_i^b))$. In the case of “*aa*|*bb*”, we assume the read coverage $c(s_j^a)$ (or $c(s_j^b)$) on $s_j^a$ (or $s_j^b$) is Bionomial distributed with parameters $N=c(s_j^a) + c(s_j^b)$ and $p=p_i^a$ (or $p_i^b$). With this assumption, the log-likelihood is calculated as $l(aa|bb)=c(s_j^a)log(p_i^a)+c(s_j^b)\log (p_i^b)$. We assign these two terms $c(s_j^a)\log (p_i^a)$ and $c(s_j^b)log(p_i^b)$ to edges $(s_i^a, s_j^a)$ and $(s_i^b, s_j^b)$ respectively. Since the Hi-C read coverage is usually biased by many factors such as GC-content, mappability and the density of restriction sites, these two terms are normalized by the total coverage $c(s_j)$ of $s_j$ to transfer absolute coverages to proportions. Since the normalized terms $p(s_j^a)log(p_i^a)$ and $p(s_j^b)\log (p_i^b)$ are both negative, to reduce the complexity of computation in later steps, reciprocals of opposite numbers of them $w_i(s_i^a, s_j^a)=-1/[p(s_j^a)log(p_i^a)]$ and $w_i(s_i^b, s_j^b)=-1/[p(s_j^b)\log (p_i^b)]$ are used as components of weights $w(s_i^a, s_j^a)$ and $w(s_i^b, s_j^b)$. On the other hand, by estimating the log-likelihood of “*aa*|*bb*” given the read coverage proportions $p_j^a$ and $p_j^b$ of $s_j^a$, $s_j^b$, the other components $w_j(s_i^a, s_j^a)$ and $w_j(s_i^b, s_j^b)$ can be calculated in the same way. Thus, the weights of edges $(s_i^a, s_j^a)$ and $(s_i^b, s_j^b)$ are the summation of two components as $w(s_i^a, s_j^a)=w_i(s_i^a, s_j^a)+w_j(s_i^a, s_j^a)$ and $w(s_i^b, s_j^b)=w_i(s_i^b, s_j^b)+w_j(s_i^b, s_j^b)$. With the same approach, the weights $w(s_i^a, s_j^b)$ and $w(s_i^b, s_j^a)$ of other two edges can be obtained by calculating the likelihood of the alternative haplotypes “*ab*|*ba*”. We normalize these four weights to make sure their summation to be 1. We measure the reliability of the edges between $s_i$ and $s_j$ by comparing “*aa*|*bb*” supporting weight summation $w(s_i^a, s_j^a)+w(s_i^b, s_j^b)$ with “*ab*|*ba*” supporting weight summation $w(s_i^a, s_j^b)+w(s_i^b, s_j^a)$. If the ratio of the smaller summation and larger summation exceeds some threshold (e.g, 0.4), these four edges are all removed from *G*. For two adjacent SNPs $s_i$ and $s_j$ belonging to the same block, we create two edges in *G* connecting the vertices known to be on the same alleles with infinite weights.

Once the CMG *G* is built, we assemble haplotypes and fill the gaps. In each connected component $G_i$ of *G* obtained by Breadth First Search (BFS)^21^, vertices are partitioned into two groups (two haplotypes) by cutting a subset of edges, satisfying that each pair of vertices corresponding to same SNP much be assigned to different groups. Since there could be many feasible solutions, according to maximum parsimony strategy, we pick the partition which cuts a subset of edges with minimum total weights. With this approach, the assembled haplotypes will not be conflict with the any of the old haplotype fragments, due to the infinite weight assignments to edges between vertices known to be in the same haplotype. This problem can be seen as a variant of the regular Minimum s-t cut problem in graph theory, and thus we call it Minimum Multiple s-t Cut problem. The formal definition is as follows.

##### **Definition 1**

(Minimum Multiple s-t Cut *problem*) Input: A weighted undirected graph $G=(V, E)$, with *V* be a set of *n* pairs of vertices. Output: A minimum cut *S*, that is, a partition of the nodes of *G* into *S* and $V\setminus S$ such that (1) exactly one of each pair of vertices belongs to *S*, (2) the total weights of the edges going across the partition is the minimum among all the partitions of the nodes satisfying (1).

In Theorem 1 below, we show that the Minimum Multiple s-t Cut problem is NP-hard by reduction from Max-Cut problem with non-negative edge weights which is known to be NP-hard^22^. In Max-Cut problem, we are given a weighted undirected graph, and we need to find a cut whose total weight is maximum among all feasible cuts.

##### **Theorem 1**

Min-Multi-Cut* is NP-hard*.

##### *Proof*

Given an instance $G' = (V', E')$ of Max-Cut with non-negative edge weights, we build an instance $G = (V, E)$ of Min-Multi-Cut as follows. For each vertex $v'_i \in V'$, create a pair of vertices $v_i^a$ and $v_i^b$ in *V*. For each edge $(v'_i, v'_j) \in E'$ ($i < j$) with weight *w*, create an edge $(v_i^a, v_j^b)$ in *E* with weight *w*. Then it’s easy to see that a Max-Cut solution $\{S', V'-S'\}$ to $G'$ is equivalent to a Min-Multi-Cut solution $\{S, V-S\}$ to *G* with $S = \{v_i^a \mid v'_i \in S'\} + \{v_i^b \mid v'_i \in V'-S'\}$, and a Min-Multi-Cut solution $\{S, V-S\}$ to *G* is equivalent to a Max-Cut solution $\{S', V'-S'\}$ to $G'$ with $S' = \{v'_i \mid v_i^a \in S\}$. $\square$

Given the complexity of Min-Multi-Cut problem, we revise one of the most popular heuristic algorithm for regular Min-Cut problem called Karger’s algorithm^23^ to solve it. Instead of determining the minimum cut for all pairs of vertices at same time, the revised Karger’s algorithm iteratively determine the cut for two pairs of vertices at each time. We decide the order of the two pairs picked by an association graph *A* built from original graph *G*. The association graph is an undirected graph in which each vertex represents a pair of vertices in *G* and an edge indicates there are at least one edge between the two pairs of vertices in *G*. For an edge (*u*, *v*) in *A* which connects two pairs of vertices $u_a$, $u_b$ and $v_a$, $v_b$ in *G*, the weight $w_A(u, v)$ is calculated as follow.

$$\begin{aligned} w_A(u, v)=1/(-r_1\log r_1 - r_2\log r_2) \end{aligned}$$

where

$$\begin{aligned} r_1=\frac{(w_G(u_a, v_a)+w_G(u_b, v_b))}{(w_G(u_a, v_a)+w_G(u_b, v_b)+w_G(u_a, v_b)+w_G(u_b, v_a))} \end{aligned}$$

and $r_2=1-r_1$ are the proportions of weights supporting two feasible cut “$u_a,v_a/u_b,v_b$” and “$u_a,v_b/u_b,v_a$” in *G* respectively. If one or more of these four edges don’t exist in *G*, the weights of missing edges are used as 0. With association graph *A*, our heuristic algorithm can be carried out by iteratively randomly picking an edge from *A* with probabilities proportional to the edges weights and determine the cut for the corresponding two pairs of vertices in *G*. After each iteration, the association graph is updated by removing this edge picked and merging the two vertices. Since the edge weights in *A* represent the reliability, this process is in accordance with the intuition that the two pairs whose cut can be determined more reliably should be picked earlier.

We repeat this process for *M* times and pick the best solution, where *M* is a parameter specified by user which represents a tradeoff between the goodness of solution and running speed.

In this algorithm, the time efficiency directly affects the goodness of solution. To speed up, instead of sampling edge and merging vertices at each time in association graph, we generate a random permutation of edges with probabilities proportional to edge weights at once. Then the edges are picked in order of permutation to generate the cut. With the use of disjoint set^21^ data structure, the whole algorithm takes $O((|E|+|V|)M)$ time.

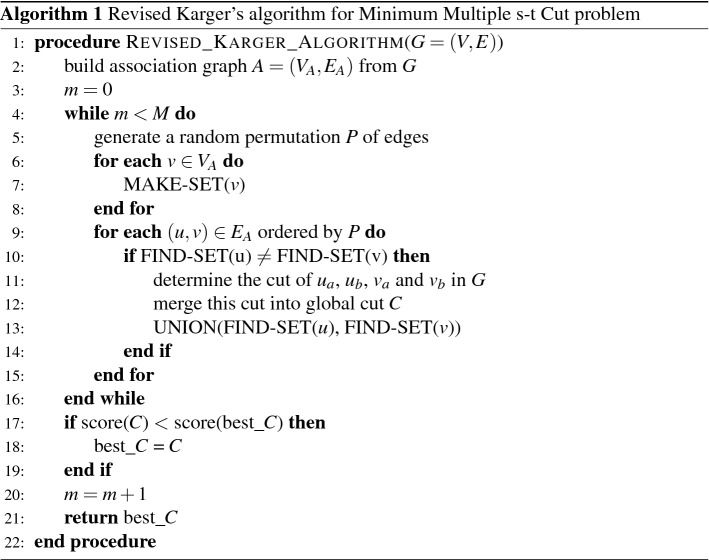



#### Gap filling in balanced allelic copy number regions using linkage disequilibrium information

After assembling and gap filling haplotypes with coverage information, there are still some tiny haplotype fragments not merged into main haplotypes yet. These tiny fragments mostly belong to the genomic regions with balanced copy numbers on two alleles, so that the coverage information fails to merge them to main haplotypes.

For these regions, we leverage the linkage disequilibrium information. The main haplotypes of each continuous non-LOH region are chosen as the cluster center, and the tiny fragments are assigned to the closest cluster according to the genomic distances. In each cluster, the main haplotypes are used as “seed haplotypes” to guide the phasing of the whole cluster by Beagle (v5.1) software^24^ with linkage disequilibrium information learned from a population of haplotypes generated by 1000 Genomes Project.

### Step4: completing

Finally, we complete the reconstruction of chromosome-level haplotypes by connecting the sequences of LOH regions with haplotypes in non-LOH regions. This process is not trivial because a chromosome may contain multiple continuous LOH and non-LOH regions, and for every pair of LOH and non-LOH regions, one of the two haplotypes of the non-LOH regions need to be decided at the same allele as the LOH sequence. Intuitively, among all the possibilities of the chromosome-level haplotypes, the one with the most supporting Hi-C paired-end reads should be chosen. For each chromosome, we use a graph model in which each vertex indicates a LOH region or a haplotype of a non-LOH region. Between every pair of LOH vertex and a haplotype vertex, there is an edge with weight representing the number of paired-end reads with one end on the LOH region and the other end on the SNPs of the haplotype. The graph needs to be partitioned into two subgraphs (as two chromosome-level haplotypes) by removing a subset of edges which satisfies (1) two haplotype vertices of the each non-LOH region belong to different subgraphs (2) the total weights of the removed edges are minimum among all the partitions satisfying (1). Since the graph is small (fewer than 10 vertices) in most cases, all feasible solutions are enumerated and the optimal solution is obtained.

## Results

We tested the performance of HiCancer in K562^25^ and KBM-7^26^ which are two human immortalized chronic myelogenous leukemia (CML) cell lines. K562 was derived from a 53-year-old Caucasian female in 1970 and KBM-7 was from a 39-year-old man. Although the two cell lines were both from CML patients, their genomes are significantly different. According to the previous study, K562 genome is near-triploid and most genomic regions are non-LOH, while KBM-7 genome is near-haploid^25,26^.

### Experimental results in K562 cell line

We chose K562 cell line because it has the highest quality phased haplotype sequences and detected LOH regions available which can be used as “ground truth” among the widely studied cancer genomes^8^. Nevertheless, the “ground truth” K562 haplotypes still have at least two shortcomings. First, the haplotypes in LOH regions are generated in the same way as non-LOH regions, which is incorrect in our opinion. Second, the haplotypes lose a large number of input SNPs and only 15 chromosomes have high-quality haplotypes availiable. However, we still believe the “ground truth” haplotypes of the 15 chromosomes are able to prove the high correctness of HiCancer if the HiCancer haplotypes in non-LOH regions processed by removing SNPs not existing in “ground truth” haplotypes are highly consistent with the “ground truth” haplotypes. We first tested the correctness and completeness of haplotypes in non-LOH regions. Then we verified the accuracy of LOH regions detected. Overall, the chromosome-level haplotypes generated by HiCancer are tested.

For convenience of comparison with “ground truth” haplotypes, the input SNPs were also downloaded from^8^. The *in situ* Hi-C reads in K562 were downloaded from^27^ and reference genome (hg19) was downloaded from UCSC Genome Browser website (https://genome.ucsc.edu/). When running HiCancer, all the built-in tools including BWA^20^, HAPCUT2^17^ and Beagle (v5.1)^24^ were all run with default parameters. The threshold of MAPQ for filtering alignments was set to be 30. All the other parameters were default (given in parenthesis in “Methods” section).

#### HiCancer can phase cancer chromosomes correctly and completely in K562 cell line

In this section, we tested the performance of HiCancer in terms of completeness and correctness by comparing with HAPCUT2 and WhatsHap^28^ which are two most widely-used phasing tools using paired-end short reads (although WhatsHap is not specially designed for Hi-C data). Since HAPCUT2 and WhatsHap do not deal with LOH regions and thus are not able to generate chromosome-level haplotypes for many chromosomes of K562, to be fair, we only focus on the non-LOH regions in comparison. Two measurements were used to evaluate the completeness of non-LOH haplotypes for each chromosome. The first one was the number of phased large haplotype blocks with at least 100 SNPs in non-LOH regions, and the second one was the proportion of non-LOH SNPs contained in these large haploytpe blocks in total. To evaluate the correctness of haplotypes, absolute error rate (AER) of the largest phased block of each chromosome was used. The calculation of AER is as follows. For one chromosome, let *S* be the intersection of SNPs in HiCancer haplotypes and “ground truth” haplotypes. For each SNP *s* in *S*, let $s_a^{phased}$ and $s_b^{phased}$ be the two alleles of *s* on the phased haplotypes, and $s_a^{truth}$ and $s_b^{truth}$ be the two alleles of *s* on the “ground truth” haplotypes. After obtaining the count $count^o$ of SNPs in *S* with $(s_a^{phased}, s_b^{phased}) = (s_a^{truth}, s_b^{truth})$ and count $count^r$ of SNPs in *S* with $(s_a^{phased}, s_b^{phased}) = (s_b^{truth}, s_a^{truth})$, the AER is calculated as

$$\begin{aligned} AER = \frac{\min \{count^o, count^r\}}{count^o + count^r}. \end{aligned}$$

In this experiment, HAPCUT2 and WhatsHap were run with default parameters.

Table 1 shows that HiCancer and HAPCUT2 both are able to phase the non-LOH regions of the vast majority of chromosomes into one single fragment of haplotypes. On chromosome 9 and 14, they both provide two large fragment of phased haplotypes because these two chromosomes both contain very long LOH regions between non-LOH regions. Chromosome 3 and X do not need to be phased because the whole chromosomes are LOH regions (see Table 2 for details). Compared with HAPCUT2 which generates haplotypes with about 70-80% of SNPs, HiCancer is able to phase haplotypes with 100% or almost 100% SNPs. With the step of completing, HiCancer is able to generate a complete single pair of haplotypes for every chromosome. At the same time, HiCancer outperforms HAPCUT2 in correctness on 12 of the 15 chromosomes with “ground truth” haplotypes available. On the other 3 chromosomes, HiCancer gives comparable correctness. On chromosome 7, HAPCUT2 generates haplotypes with extremely high AER (46.8894%) caused by a switching error appearing at the middle of the chromosome. HiCancer successfully corrects the switching error and reduces the AER to 1.5327% which is at the same level as the AER of other chromosomes. Compared with HAPCUT2 and HiCancer, WhatsHap is only able to generate short haplotype fragments, which lead to the extremely low completeness in the whole genome and high accuracy in some chromosomes.

**Table 1:** Comparing the phasing performance of HiCancer with HAPCUT2 and WhatsHap on K562 genome.

Chrom	# large blocks (>100 SNPs) in non-LOH regions				% non-LOH SNPs in large blocks			AER of largest block		
	HAPCUT2	WhatsHap	HiCancer−	HiCancer	HAPCUT2	WhatsHap	HiCancer−	HAPCUT2	WhatsHap	HiCancer−
1	1	1	1	1	81.6848%	0.0699%	**100%**	1.1594%	38.6796%	**0.5679%**
2	1	0	1	1	76.3653%	0	**100%**	5.3739%	**0**	4.7767%
3	N/A	N/A	N/A	1	N/A	N/A	N/A	N/A	N/A	N/A
4	1	12	1	1	72.4314%	2.2483%	**100%**	1.3834%	7.1174%	**1.0614%**
5	1	3	1	1	75.6427%	0.6845%	**100%**	0.82224%	5.2632%	**0.5879%**
6	1	25	1	1	78.5579%	4.6763%	**100%**	3.5155%	29.1116%	**1.4087%**
7	1	2	1	1	80.8724%	0.3504%	**100%**	46.8894%	4.4456%	**1.5327%**
8	1	1	1	1	77.2177%	0.1241%	**100%**	0.78361%	**0**	0.6451%
9	2	0	2	1	89.9577%	0	**99.5765%**	N/A	N/A	N/A
10	1	1	1	1	82.1379%	0.2219%	**99.9148%**	2.6609%	**1.3605%**	2.5126%
11	1	7	1	1	77.1570%	1.7480%	**100%**	1.2725%	**0**	0.6315%
12	1	1	1	1	78.4648%	0.1580%	**100%**	0.6190%	**0**	0.5313%
13	1	0	1	1	86.2647%	0	**100%**	N/A	N/A	N/A
14	2	1	2	1	67.2566%	17.4165%	**100%**	N/A	N/A	N/A
15	1	0	1	1	80.9122%	0	**100%**	0.9005%	**0**	0.7181%
16	1	2	1	1	85.7108%	0.5946%	**100%**	**1.2779%**	5.9575%	1.3441%
17	1	7	1	1	83.3136%	5.4616%	**100%**	**0.5356%**	46.5448%	0.6016%
18	1	1	1	1	71.7267%	0.2081%	**100%**	N/A	N/A	N/A
19	1	1	1	1	87.1062%	0.2503%	**100%**	0.7356%	27.2727%	**0.6443%**
20	1	1	1	1	84.6577%	0.4818%	**100%**	**0.2465%**	2.4591%	0.3847%
21	1	2	1	1	81.7515%	0.7517%	**100%**	N/A	N/A	N/A
22	1	0	1	1	92.9897%	0	**99.9742%**	N/A	N/A	N/A
X	N/A	N/A	N/A	1	N/A	N/A	N/A	N/A	N/A	N/A

“Chrom” is the abbreviation of “Chromosome”; “HiCancer−” represents the output of HiCancer without doing the step of completing. Numbers in boldface highlight the best completeness and correctness. For the chromosomes whose whole or almost whole chromosomes are LOH regions, ‘N/A’ is used for some statistics cannot be calculated.

#### HiCancer can detect LOH regions accurately in K562 cell line

We also tested the accuracy of LOH regions detected by HiCancer. LOH regions detected by 10x genomics reads in^8^ were used as “ground truth”. The accuracy was evaluated by the precision and sensitivity calculated as follows. For each chromosome, we obtained the total length “length_call” of detected LOH regions , total length “length_truth” of LOH regions in “ground truth” and the length “length_overlap” of their overlaps. The precision is the ratio of “length_overlap” and “length_call” , and sensitivity is the ratio of “length_overlap” and “length_truth”.

Observed from Table 2 that HiCancer detects LOH regions with high accuracy. On the 11 chromosomes with total length of LOH region higher than 4Mb in “ground truth”, HiCancer obtains sensitivities from 94.4 to 100% and most of them are higher than 98%. On 10 of these 11 chromosomes, the precisions are from 70.2 to 83.0% and the precision of chromosome X is 50.9%. For the whole genome, the precision is 70.58% and sensitivity is 98.22%. We further investigated the reason that the precisions are not as high as sensitivities. We found that on many chromosomes such as chromosome 3, 9, 13, 14, 22 and X, HiCancer treats almost the all regions as LOH while the “ground truth” only treats parts of the chromosomes as LOH regions. However, from Figure 1 of their paper^8^, it is easy to see that almost all regions of these chromosomes are short of heterozygous SNPs. We believe this is caused by the different definitions of LOH regions. Some regions with very small number of heterozygous SNPs were seen as non-LOH regions in^8^ but detected by HiCancer as LOH regions because they should not be treated as two allele when building chromosome-level haplotypes.

**Table 2:** Performance of LOH detection of HiCancer on K562 genome.

Chrom	# LOH	# non-LOH	Calls (bp)	Ground truth (bp)	True positive (bp)	Precision	Sensitivity
1	0	1	0	0	0	N/A	N/A
2	4	4	59,000,000	43,080,000	41,440,000	70.2373%	96.1931%
3	2	1	196,000,000	143,160,000	143,160,000	73.0408%	100%
4	1	2	3,000,000	3,200,000	2,480,000	82.6667%	77.5000%
5	3	4	4,000,000	0	0	0	N/A
6	2	3	3,000,000	0	0	0	N/A
7	0	1	0	0	0	N/A	N/A
8	0	1	0	0	0	N/A	N/A
9	8	7	103,000,000	77,080,000	73,880,000	71.7282%	95.8485%
10	1	1	47,000,000	36,400,000	35,640,000	75.8298%	97.9121%
11	0	1	0	0	0	N/A	N/A
12	4	4	26,000,000	21,600,000	20,400,000	78.4616%	94.4444%
13	3	3	92,000,000	77,640,000	76,360,000	83.0000%	98.3514%
14	1	1	86,000,000	69,240,000	68,280,000	79.3953%	98.6135%
15	0	1	0	0	0	N/A	N/A
16	0	1	0	40,000	0	N/A	0
17	2	2	22,000,000	17,600,000	17,040,000	77.4545%	96.8182%
18	0	1	0	0	0	N/A	N/A
19	0	1	0	0	0	N/A	N/A
20	1	1	26,000,000	19,920,000	19,840,000	76.3077%	99.5984%
21	0	1	0	0	0	N/A	N/A
22	1	1	28,000,000	22,120,000	21,720,000	77.5714%	98.1917%
X	2	2	151,000,000	76,880,000	76,880,000	50.9139%	100%
Total	35	45	846,000,000	607,960,000	597,120,000	70.5816%	98.2170%

Numbers in boldface highlight the best completeness and accuracy. “Chrom” is the abbreviation of “Chromosome”; “Calls”, “Ground truth” and “True positive” represent the total length of called LOH regions, total length of LOH regions in “ground truth” and their overlapped length respectively; “# LOH” and “# non-LOH” represent the numbers of continuous LOH and non-LOH regions respectively.

### Experimental results in KBM-7 cell line

In addition to K562 cell line, we also tested the performance of HiCancer in KBM-7 cell line. The Since KBM-7 cell line does not have high-quality phased haplotypes to verify the correctness, we only tested the completeness using the same criteria in K562 experiments. The *in situ* Hi-C reads in K562 were downloaded from^27^ and reference genome (hg19) was downloaded from UCSC Genome Browser website (https://genome.ucsc.edu/). BWA mem and GATK HaplotypeCaller (v4.1) were used to map Hi-C reads and call the SNPs respectively, both with default parameters. HiCancer, HapCut2 and WhatsHap were all run with default parameters. The default parameters of HiCancer are given in parenthesis in “Methods” section.

Table 3 shows that, same as K562 cell line, HiCancer generates complete chromosome-level haplotypes with almost all SNPs in KBM-7 cell line. However, the results of HapCut2 and WhatsHap are different from those in K562 cell line in two aspects. First, the “% non-LOH SNPs in large blocks” values of HapCut2 fluctuate from 35.1531 to 99.0394% while the values are mostly around 80% in K562. Second, the “% non-LOH SNPs in large blocks” values of WhatsHap are significantly higher than those in K562, although they are still much lower than HapCut2 and HiCancer. We believe these differences result from the low proportion of non-LOH regions in KBM-7 genome.

**Table 3:** Comparing the phasing performance of HiCancer with HAPCUT2 and WhatsHap on KBM7 genome.

Chrom	# large blocks (>100 SNPs) in non-LOH regions				% non-LOH SNPs in large blocks		
	HAPCUT2	WhatsHap	HiCancer−	HiCancer	HAPCUT2 (%)	WhatsHap (%)	HiCancer− (%)
1	2	12	2	1	77.6763	50.5983	**100**
2	2	14	2	1	81.4451	48.9940	**100**
3	1	3	1	1	95.3980	66.7125	**100**
4	2	2	2	1	85.3083	29.5084	**100**
5	1	1	1	1	63.9966	14.2653	**100**
6	1	2	1	1	99.0394	61.8098	**100**
7	2	10	2	1	75.8405	51.0057	**100**
8	1	105	1	1	82.3343	19.3996	**100**
9	1	5	1	1	58.9197	39.0244	**100**
10	2	7	2	1	81.3601	42.8793	**100**
11	1	3	1	1	80.0613	41.4443	**100**
12	1	2	1	1	35.1531	12.0673	**99.4578**
13	3	3	3	1	81.3990	50.3092	**100**
14	1	3	1	1	43.2377	11.7647	**99.8856**
15	1	27	1	1	80.6579	25.0341	**100**
16	1	14	1	1	87.5469	62.1534	**100**
17	1	1	1	1	98.2761	62.1605	**100**
18	1	1	1	1	65.0336	17.6683	**100**
19	1	9	1	1	71.7284	31.3807	**100**
20	1	9	1	1	95.0443	73.8740	**100**
21	2	4	2	1	92.0796	81.5865	**100**
22	1	4	1	1	49.7429	38.3081	**99.5732**

“Chrom” is the abbreviation of “Chromosome”; “HiCancer−” represents the output of HiCancer without doing the step of completing. Numbers in boldface highlight the best completeness.

## Discussion

We presented HiCancer, a new computational pipeline for phasing cancer genome with Hi-C reads. We found that HiCancer is able to generate chromosome-level haploytypes for cancer genome with very high completeness and correctness using only Hi-C paired-end reads.

There are a number of areas that our methods and approaches can be further improved. First, the current version of HiCancer needs SNPs called as input. Since the SNP list is not available for all cancer genome, it limits the application of HiCancer. It remains to be explored to improve HiCancer by adding a step of SNP calling using Hi-C reads. The difficulty is that the Hi-C read coverage is greatly affected by factors such as mappability, GC content and density of restriction sites which may reduce the accuracy of called SNPs. But we believe the appropriate normalization will be able to solve this problem. Second, HiCancer can only phase cancer genome using SNPs as markers for now, but the future work needs to be done to add the function of dealing with small insertions and deletions. Third, HiCancer only generates two haplotypes for each chromosome now, but future work is required to further distinguish multiple copies of each haplotype according to somatic mutations. Due to the complexity of cancer genome, this could be too challenging using only Hi-C reads. However, by combining Hi-C reads with the newest third-generation sequencing long reads such as Pacbio HiFi reads (high-fidelity long reads) and Oxford Nanopore ultra-long reads, we believe there is a chance to solve this problem and generate complete cancer genome map.

## Data Availability

The source code of HiCancer can be accessed at: https://github.com/alanpwhhero/HiCancer
